# Inhibition shapes selectivity to vocalizations in the inferior colliculus of awake mice

**DOI:** 10.3389/fncir.2012.00073

**Published:** 2012-10-11

**Authors:** Zachary M. Mayko, Patrick D. Roberts, Christine V. Portfors

**Affiliations:** ^1^School of Biological Sciences, Washington State UniversityVancouver, WA, USA; ^2^Department of Biomedical Engineering, Oregon Health and Science UniversityPortland, OR, USA

**Keywords:** inferior, colliculus, mouse, frequency tuning, inhibition, vocalization

## Abstract

The inferior colliculus (IC) is a major center for integration of auditory information as it receives ascending projections from a variety of brainstem nuclei as well as descending projections from the thalamus and auditory cortex. The ascending projections are both excitatory and inhibitory and their convergence at the IC results in a microcircuitry that is important for shaping responses to simple, binaural, and modulated sounds in the IC. Here, we examined the role inhibition plays in shaping selectivity to vocalizations in the IC of awake, normal-hearing adult mice (CBA/CaJ strain). Neurons in the IC of mice show selectivity in their responses to vocalizations, and we hypothesized that this selectivity is created by inhibitory microcircuitry in the IC. We compared single unit responses in the IC to pure tones and a variety of ultrasonic mouse vocalizations before and after iontophoretic application of GABA_A_ receptor (GABA_A_R) and glycine receptor (GlyR) antagonists. The most pronounced effects of blocking GABA_A_R and GlyR on IC neurons were to increase spike rates and broaden excitatory frequency tuning curves in response to pure tone stimuli, and to decrease selectivity to vocalizations. Thus, inhibition plays an important role in creating selectivity to vocalizations in the IC.

## Introduction

Neural processing of sensory information relies on the microcircuitry and cellular properties of neurons in the sensory pathway. Identifying the microcircuitry in specific sensory nuclei is important for understanding how behaviorally relevant information is processed, and for determining how changes in cellular properties caused by neural modulators alters sensory processing. Within the auditory pathway, microcircuitry is well understood in the cochlear nucleus (Young and Oertel, 2010) and the nucleus laminaris in chick (Wang et al., 2010). The necessary characterization of microcircuitry has not yet been completed in the main auditory midbrain nucleus, the inferior colliculus (IC). This is a necessary and critical step for a full understanding of the IC's function in analyzing and identifying complex and behaviorally relevant auditory signals.

The IC is the major processing and integrating center in the auditory midbrain (Winer and Schreiner, 2005) as it receives massive ascending projections from all auditory brainstem nuclei (Adams, 1979; Brunso-Bechtold et al., 1981; Frisina et al., 1998) as well as descending projections from the auditory thalamus and cortex (Saldana et al., 1996; Winer et al., 1998). In addition, there are commissural (Aitkin and Phillips, 1984) and intrinsic (Oliver et al., 1991) projections. Ascending projections into the IC are glutamatergic, GABAergic, or glycineric (Willard and Ryugo, 1983; Saint Marie and Baker, 1990; Saint Marie, 1996; Cant, 2005; Schofield, 2005) and commissural projections are GABAergic (Reetz and Ehret, 1999). This convergence of excitatory and inhibitory inputs onto single neurons in the IC results in microcircuits that are important for regulating response properties. In particular, inhibitory inputs play an important role in shaping IC responses to simple, binaural, and modulated sounds (Faingold et al., 1989, 1991; Vater et al., 1992a; Yang et al., 1992; Park and Pollak, 1993, 1994; Casseday et al., 1994; Klug et al., 1995; Fuzessery and Hall, 1996; Le Beau et al., 1996; Palombi and Caspary, 1996; Burger and Pollak, 1998; Koch and Grothe, 1998; LeBeau et al., 2001; Caspary et al., 2002; Zhang and Kelly, 2003). There has been much less focus, however, on how inhibitory inputs to IC neurons shape responses to more complex sounds such as social vocalizations.

In the Mexican free-tailed bat, inhibition plays a role in creating selectivity to social vocalizations (Klug et al., 2002; Xie et al., 2005). Pharmacologically blocking GABAergic and glycinergic receptors in the IC decreases selectivity to social vocalizations (Klug et al., 2002; Xie et al., 2005). In contrast, blocking inhibition in the nuclei of the lateral lemniscus does not alter the selectivity of neurons to social vocalizations (Xie et al., 2005). These results indicate that inhibitory circuitry in IC is important for creating selectivity for social vocalizations in bats (Xie et al., 2005). It is not known in any other species, however, whether inhibitory microcircuits in the IC function in the same manner as in bats to create selectivity for vocalizations. Determining whether this is a general feature or one that is a specialization of bats is crucial for understanding the evolution of neural processing of communication sounds.

The purpose of this study was to examine how inhibitory microcircuitry in the IC shapes selectivity to vocalizations in awake, normal-hearing adult mice (CBA/CaJ strain). Selectivity to social vocalizations occurs in the IC of mice, and just like in bats, there is heterogeneity in the level of selectivity with some neurons being highly selective for one or two vocalizations and other neurons responding to many vocalizations (Portfors et al., 2009; Holmstrom et al., 2010). Because mice are becoming an important model for understanding neural mechanisms of auditory processing disorders due to the benefits of genetic engineering, a thorough understanding of IC structure and function in normal hearing mice is necessary.

We locally blocked inhibitory inputs to IC neurons by iontophoretically applying antagonists to GABA_A_ receptors (GABA_A_R) and glycine receptors (GlyR). We compared responses to tones and vocalizations before and after application of these antagonists. We used the changes in tone responses to identify potential ways that inhibition could shape selectivity to vocalizations. We found that blocking inhibitory receptors increased evoked firing in all neurons and changed the shape of the excitatory receptive field in some neurons. Both of these effects contributed to the decreased selectivity to vocalizations. These results suggest that the complex interplay between excitation and inhibition in the IC helps create heterogeneous neural responses to behaviorally relevant sounds.

## Materials and methods

We recorded auditory responses from single neurons in the IC of awake, restrained CBA/CaJ mice. All mice were female and between the ages of 2 and 12 months. The CBA/CaJ strain exhibits normal hearing sensitivity into its second year of life (Willott, 1986, 1991, 2005). As in our previous studies with this strain, we did not find any apparent differences in neural response properties across the ages of mice used in this study (Portfors and Felix II, 2005; Portfors et al., 2009, 2011). Animals were housed with same-sex litter mates on a reversed 12 h light/dark schedule. All mice had *ad libitum* access to food and water. All animal care and experimental procedures were in accordance with the guidelines of the National Institutes of Health, and were approved by the Washington State University Institutional Animal Care and Use Committee.

### Surgical procedures

At least 24 hours prior to the first electrophysiological recording session, we mounted a headpost onto the skull of the mouse (Muniak et al., 2012). We placed the mouse in an induction chamber with isoflurane to induce anesthesia. We then placed it in a rodent stereotaxic frame with a mouse adaptor and maintained isoflurane inhalation via a nose mask. We made an incision in the scalp along the midline and reflected the skin laterally. We cemented a hollow metal rod (the headpost) onto the skull and a tungsten ground electrode into the right cerebral cortex using ultraviolet-cured dental cement. Using stereotaxic coordinates slightly modified from Paxinos and Franklin (2001), we made a craniotomy (usually 1 × 1 mm) over top of the left IC. We then covered the hole with petroleum jelly or bone wax to prevent the brain from dehydrating, applied a local anesthetic (lidocaine) and an antibiotic (Neosporin) to the exposed muscle, and returned the mouse to its home cage to recover from surgery.

### Acoustic stimulation

Acoustic stimulation was computer-controlled and included tone bursts (100 ms duration, 1 ms rise/fall time, 4/s) and a suite of mouse vocalizations used in a previous study of mouse IC (Portfors et al., 2009). All stimuli were stored in the computer and were output through a high speed, 16-bit digital-to-analog converter (Microstar Laboratories, Bellevue, WA, USA; 400,000 samples/s), fed to a programmable attenuator (Tucker Davis Technologies, Alachua, FL, USA; PA5), a power amplifier (Parasound), and to a leaf tweeter speaker (Emit) located 10 cm away from the mouse. We tested the acoustic properties of the system using a 1/4-inch calibrated microphone (Bruel and Kjaer, Denmark; model 4135) placed in the position normally occupied by the animal's ear. There was a smooth, gradual decrease in sound pressure from 6 to 100 kHz of about 3 dB per 10 kHz. Distortion components in tonal stimuli were buried in the noise floor, at least 50 dB below the signal level, as measured by custom-designed software performing a fast Fourier transform of the digitized microphone signal.

### Electrophysiological recording and drug application

We conducted electrophysiological experiments in a single-walled sound-attenuating chamber. On experimental days, we placed the animal securely into a foam body mold and attached the headpost to a custom-made stereotax apparatus (Muniak et al., 2012). If at any time during the experiment the animal showed signs of distress, the experiment was terminated. Experimental sessions lasted 4–5 h and we used each animal in 1–3 sessions.

We obtained responses of single units to pure tones and mouse vocalizations before and after the application of GABA_A_R and GlyR antagonists. To obtain well isolated single unit responses, we used a single micropipette electrode mounted on a five-barreled pipette for microiontophoretic application of drugs (Havey and Caspary, 1980). The tip of the single electrode extended 10–25 μm beyond the multibarrel pipette and contained 1 M NaCl. We broke the tip of the multibarrel pipette to a diameter of approximately 30 μm. We filled the center barrel of the multibarrel pipette with 1 M NaCl and connected it to a sum channel to balance all currents used to apply or retain drugs. The rest of the barrels contained the GABA_A_R antagonists bicuculline (10 mM, pH 3.0, vehicle 0.9% physiological saline; Sigma) and the GlyR antagonist strychnine (10 mM, pH 3.0, vehicle 0.9% physiological saline; Fluka, Milwaukee, WI). We used similar iontophoresis currents for drug retention and ejection to those used in previous studies (Wenstrup and Leroy, 2001; Ingham and McAlpine, 2005; Sanchez et al., 2008). Bicuculline and strychnine were retained with negative current (−15 nA each) and ejected with positive current (range, +10 nA to +40 nA each). As in previous studies (Razak and Fuzessery, 2009), our control experiments confirmed that current injection as high as 100 nA through pH-adjusted vehicle solutions did not have any effect on neuronal discharge properties.

We prepared all drugs and recording solutions the day of the experiment. We inserted separate silver wires into each barrel of the micropipette electrode and connected them to a microiontophoresis current generator (model 650, David Kopf Instruments, Tujunga, CA) to separately control the retention and ejection currents for each drug. We advanced the electrodes into the IC using a hydraulic micropositioner (David Kopf Instruments, Tujunga, CA) located outside the acoustic chamber. Extracellular action potentials were amplified (Dagan Corporation, Mineapolis, MN, USA), filtered (bandpass, 500–6000 Hz; Krohn-Hite, Brockton, MA, USA) and sent through a spike enhancer (Fredrick Haer, Bowdoin, ME, USA) before being digitized (Microstar Laboratories, Bellevue, WA, USA; 10,000 samples/s). Neural waveforms were displayed and archived using custom-written C++ software. Waveforms, raster plots, peri-stimulus time histograms (PSTHs), and statistics were viewed on-line and stored for off-line analysis.

We used tone bursts as search stimuli (varying duration, 1 ms rise/fall time) to obtain well isolated single units. All tests were first run in the control condition. We obtained characteristic frequency (CF) and minimum threshold (MT) audiovisually. We defined CF as the frequency that evoked a response to 50% of the stimulus presentations at the lowest intensity, and MT as the lowest intensity that evoked a response 50% of the time to the CF. To obtain excitatory frequency tuning curves, we presented pure tones (100-ms duration, 1 ms rise/fall time, 4/s, 200 ms recording window) between 6 and 100 kHz in 2-kHz steps, and varied the intensity in 10- to 20-dB steps starting at threshold. We presented each frequency and intensity pair 10–20 times. We then obtained responses to vocalizations by presenting the suite of 14 vocalizations (variable duration, 1 ms rise/fall time, 4/s, 200-ms recording window) 10–40 times at multiple intensities.

Once the stimulus protocol was completed in the control condition, we applied the GABA_A_R and GlyR antagonists. Drug ejection times varied depending on the effect of the drug. We ejected bicuculline and strychnine together because we were interested in the general effects of inhibition on responses to vocalizations rather than the separate effects of GABAergic and glycinergic inhibition. We initially applied low ejection currents (+10 nA) and then gradually increased the current if there was no effect. Once the response reached a steady-state, we kept the ejection currents at this level. We then ran the same protocol as in the control condition. At the end of the stimulus protocol, we turned off the ejection current and re-applied the retention current. Complete or partial recovery was determined by comparing response rate to a CF tone in the no-drug, drug and recovery states.

### Data analysis

Spike counts and raw waveforms were stored in the computer during data collection. We examined raw waveforms off-line to ensure only spikes from well isolated single units were used in the data analysis. Single units had signal-to-noise ratios of at least 4:1 and an inter-spike interval of at least 2 ms. Data were exported from the data collection software and analyzed using programs written in Matlab (The MathWorks, Inc., Natick, MA, USA). We generated frequency tuning curves from the pure tone tests using statistical comparisons between evoked responses and spontaneous activity (Holmstrom et al., 2007), and determined sharpness of tuning by calculating Q10dB values. We compared the bandwidth of the tuning curve at the highest intensity before and after application of drugs to determine the extent of broadening. We defined broadening as an expansion of the frequency range of greater than 2 kHz.

We calculated a selectivity index value (SI) for each neuron that responded to at least one vocalization (criterion of at least 50% of presentations evoking a time-locked response or an increase in rate of 20% above spontaneous activity). We defined the SI as: SI = (*Ct* − *Ce*)/*Ct*, where *Ct* is the number of calls presented and *Ce* is the number of calls that evoked a response (at the highest intensity presented). Essentially, the SI value can be thought of as providing a normalized value to the number of vocalizations that fail to evoke a response. Thus, a SI value of 0 means that no vocalizations fail to evoke a response, and the neuron is defined as un-selective. A high SI value indicates that a large number of vocalizations do not evoke responses, thus defining the neuron as selective. The highest SI value that can be calculated is determined by the number of vocalizations presented. With our suite of 14 stimuli, the highest SI value (13 vocalizations failing to evoke a response) was 0.93. We used a standard student *t*-test (two-tailed, equal variance) to determine whether there was a significant difference between the selectivity of neurons under control and drug conditions.

To test whether changes in selectivity to vocalizations during application of GABA_A_R and GlyR antagonists were related to changes in the excitatory frequency tuning curve of the neuron, we applied a modeling methodology developed and utilized in our previous studies of IC (Holmstrom et al., 2007, 2010). For each neuron, a model was optimized to approximate the relationship between the pure tone input and the resulting firing rate of the neuron. The model was a discrete (in both frequency and time) linear finite impulse response filter. We used this model to predict how the neuron would respond to each vocalization by converting each vocalization into a spectrographic representation and convolving it with the filter. By comparing each predicted response to the actual response, we could determine how well each neuron's excitatory frequency tuning curve explained the neural responses to each vocalization.

## Results

**Figure 1:**
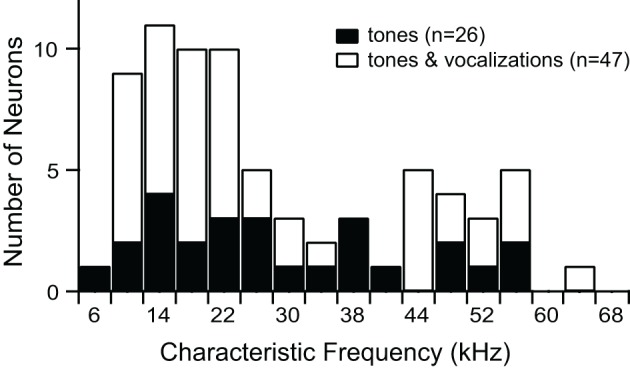
**Characteristic frequencies of single units presented with tones alone, or tones and vocalizations**.

We recorded responses of 73 single units before and after blocking GABA_A_R and GlyR. Characteristic frequencies (CFs) of the units ranged from 6 kHz to 65 kHz (Figure 1).

### Inhibition shapes responses to simple stimuli in the IC of awake mice

We obtained frequency tuning curves before and after blocking GABA_A_R and GlyR in 72 single units (1 unit was presented with vocalization stimuli but not tone stimuli). As has been shown previously in anesthetized animals (Vater et al., 1992a; Palombi and Caspary, 1996; LeBeau et al., 2001), we found that the major effect of blocking GABA_A_R and GlyR in the IC was to increase neuronal firing rates. In 3/72 neurons, the increased rate was only in spontaneous activity; no change occurred in tone-evoked responses.

**Figure 2:**
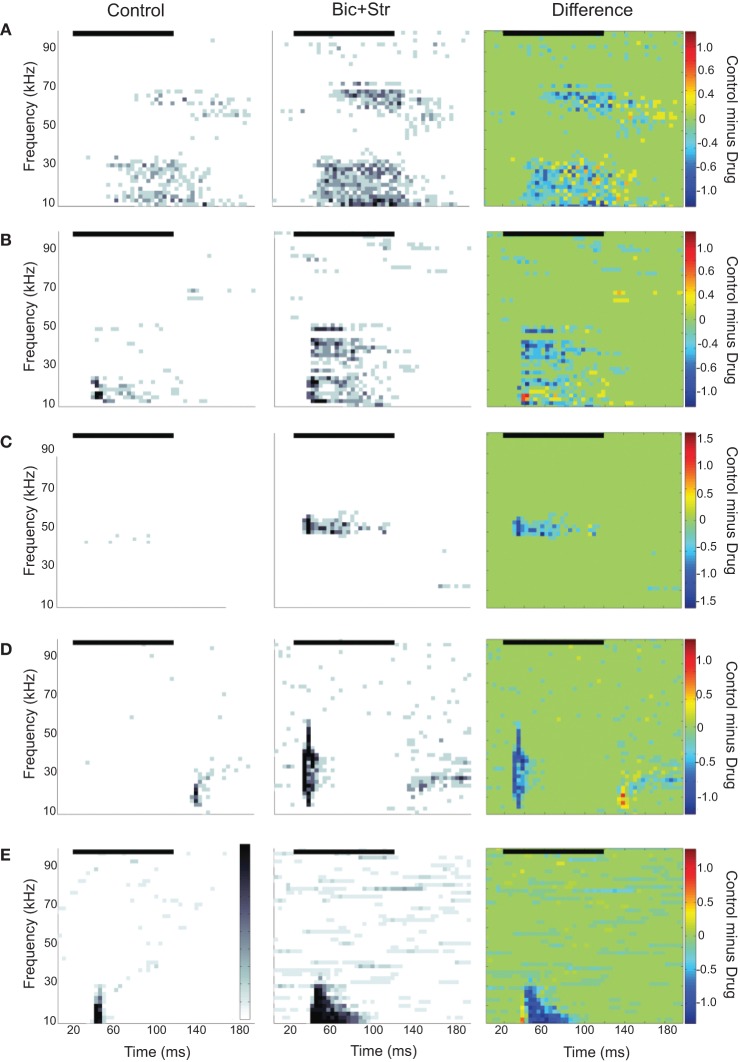
**Spectral-temporal histograms of different effects of blocking GABA_A_R and GlyR on IC responses to tones.** Each row is a different IC neuron. The order of the panels left to right is control condition, drug condition, and control-drug (difference). In the difference plots, cool colors indicate greater firing rates under drug condition and warm colors indicate higher firing rates under control conditions. **(A)** Increased evoked firing rate to tones without a broadening of the excitatory frequency tuning curve. **(B)** Increased rate and broadening of the excitatory frequency tuning curve. **(C)** Conversion of an O-shaped to a V-shaped excitatory frequency tuning curve. **(D)** Latency shift. **(E)** Conversion of an onset to a sustained evoked firing pattern. In all plots, the black horizontal line indicates the sound stimulus. Bin width is 4 ms.

We found that 35/72 neurons increased their firing rate to tones only within the control excitatory frequency tuning curve; there was no change in shape of the excitatory frequency tuning curve. Figure 2A illustrates a neuron with this type of response. The evoked responses are shown as spectral-temporal histograms in which frequency is plotted vs. time for one intensity level, and the color of each 4 ms bin represents the average number of spikes per presentation. This representation allows both spectral and temporal information to be displayed in one plot (Portfors and Roberts, 2007). The first plot shows the response under control conditions, the second plot is with iontophoretic application of bicuculline and strychnine, and the third plot is the difference between the two conditions (control condition minus drug condition, where the color scale indicates which response is stronger in each spectral-temporal bin; green indicates no difference, warm colors indicate response magnitude in control condition is greater, and cool colors indicate response magnitude in drug condition is greater). The neuron in Figure 2A had two discrete regions of excitation and was classified as a multiply-tuned (Portfors and Felix II, 2005) or type IV (Egorova et al., 2001; Portfors et al., 2011) unit. As can be seen in the spectral-temporal histogram, the response rate increased when GABA_A_R and GlyR were blocked, increasing by 68% with no broadening of the excitatory tuning curve. To quantify the changes in rate between the control and drug conditions, we subtracted the area under the control frequency tuning curve from the area under the drug frequency tuning curve for each neuron. On average, the response rate increased by 114% when GABA_A_R and GlyR were blocked.

We found that 23/72 neurons increased their firing rate and had a broadening of their V-shaped excitatory tuning curve when inhibition was blocked. Figure 2B illustrates a neuron with this type of response. The first spectral-temporal histogram shows that only a narrow range of tones at low frequencies evoked a response (Q10dB value of 1.43). The second spectral-temporal histogram shows the broader range of frequencies that evoked a response when bicuculline and strychnine were applied to the neuron. The third spectral-temporal histogram shows the difference between the control and drug conditions and clearly illustrates the range of frequencies that were suppressed by the inhibitory inputs in the control condition. The Q10dB value decreased from 1.43 to 0.78. In this example, a firing pattern change from a phasic to sustained response can also be seen.

Figure 2C illustrates another way that inhibitory inputs can shape excitatory frequency tuning curves; by suppressing responses to particular frequencies at certain intensities. Neurons affected by inhibition in this way are commonly called O-type (Ramachandran et al., 1999; Davis et al., 2003) or type II (Egorova et al., 2001). Under control conditions (first spectral-temporal histogram), responses to a CF tone were suppressed at high intensities. After GABA_A_R and GlyR were blocked, the CF tone at high intensities evoked a strong response (drug and difference spectral-temporal histograms in Figure 2C). Eleven of the neurons we recorded from had O-shaped tuning curves, and all of these changed their tuning to V-shaped when inhibition was blocked. Overall, 34 neurons showed increases in rate and changes in the shape of their excitatory tuning curve (broadening of V-shape or change from O- to V-shape) when inhibition was blocked.

We also found that inhibition can affect timing of tone-evoked responses. Figure 2D shows an example where blocking GABA_A_R and GlyR caused a large shift in latency. The median first spike latency in the control condition was 115 ms, and it decreased to 10 ms when bicuculline and strychnine were applied. A shift in latency occurred in 11 of 72 neurons (five had no change in tuning curve shape, four had broadening of their tuning curve, two changed from O- to V-shaped tuning curves). The changes in latency ranged from 5 to 105 ms. Inhibition can also influence temporal firing pattern. Figure 2E shows one neuron in which inhibition created an onset response to a CF tone. Blocking GABA_A_R and GlyR converted the onset response to sustained response. This effect occurred in 10 neurons (eight had no change in tuning curve shape, one had broadening of its tuning curve, one changed from O- to V-shaped).

### Inhibition shapes responses to vocalizations in the IC of awake mice

**Figure 3:**
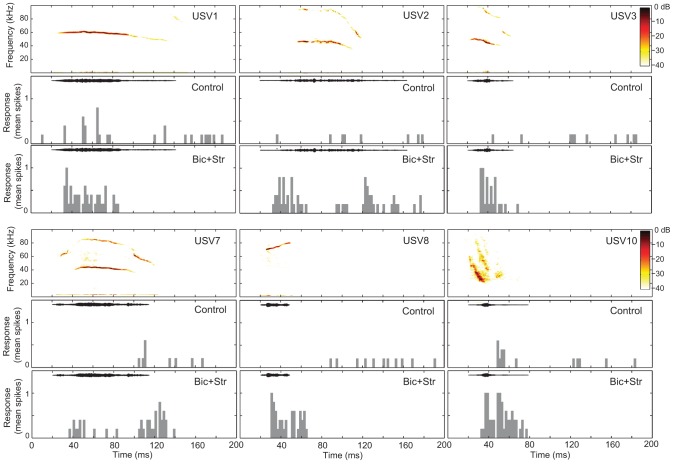
**Blocking GABA_A_R and GlyR decreases neuronal selectivity to vocalizations in the IC.** The responses of one single unit to six vocalizations before (control) and after (Bic + Str) blocking GABA_A_R and GlyR. Responses to only 6 of the 14 presented vocalizations are shown for clarity. SI values were 0.57 and 0 in the control and drug conditions, respectively. Bin width is 4 ms.

We presented vocalization stimuli to 47 single units (Figure 1). All of the vocalizations were ultrasonic (all energy above 20 kHz), but they had various spectral ranges, durations, and frequency modulations. We used this set of stimuli in a previous study of neural selectivity in the IC of mice (Portfors et al., 2009). Most neurons we recorded from had excitatory receptive areas that encompassed the spectral range of at least one of the vocalization stimuli. Forty neurons responded to at least one vocalization. We found that blocking GABA_A_R and GlyR decreased selectivity in most of these neurons (Figure 3).

**Figure 4:**
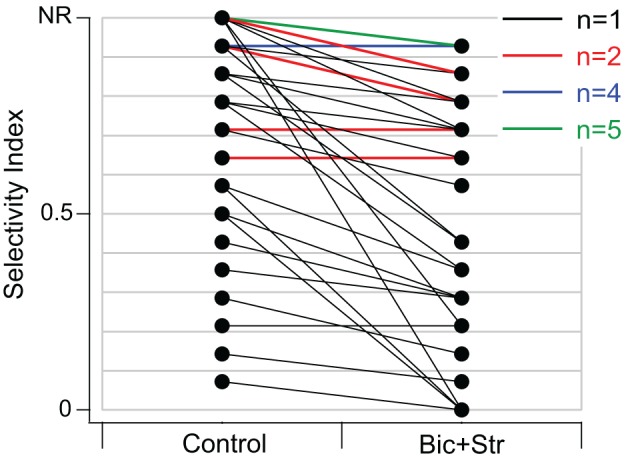
**Blocking GABA_A_R and GlyR significantly decreased selectivity to vocalizations in the IC of awake mice.** SI values in control and drug condition for each neuron. Black lines represent one single unit, red lines represent two single units with the same values, blue lines represent four units with the same values, and green lines represent five units with the same values. Neurons in the NR (no-response) category did not respond to any vocalizations in the control condition but did respond to at least one vocalization in the drug condition. Thirty-one of forty neurons had decreased SI values.

SI values significantly decreased when GABA_A_R and GlyR were blocked (*p* < 0.001). The average SI value in the control condition was 0.76 and 0.58 in the drug condition. Of the 40 neurons that responded to one or more vocalizations, 31 had decreased selectivity when bicuculline and strychnine were applied (Figure 4). Eleven neurons did not respond to any of the vocalizations under control (no response, NR, in Figure 4) but did respond to at least one vocalization during drug application.

We examined whether changes in selectivity to vocalizations were related to changes in excitatory frequency tuning curves in 39 of the 40 neurons that responded to vocalizations (1 of the 40 neurons did not have a frequency tuning curve). Of those 39 neurons, 29 showed decreases in selectivity to vocalizations.

**Figure 5:**
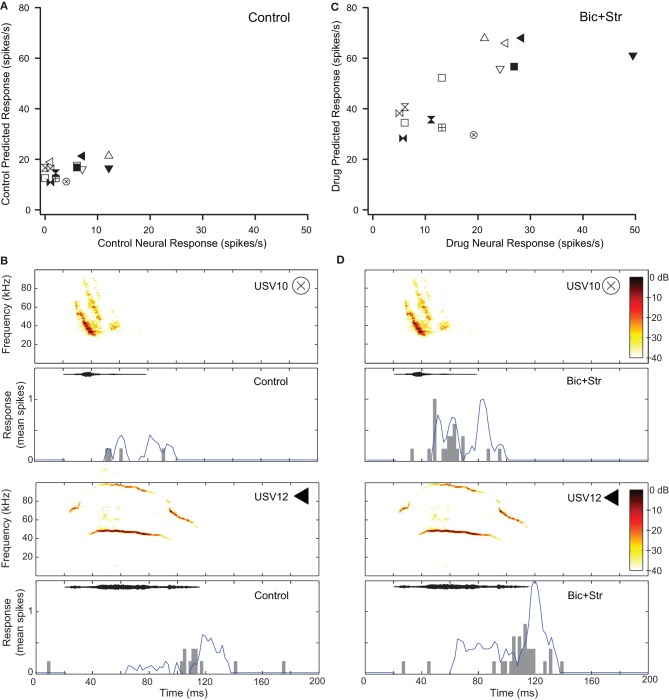
**Decreased selectivity to vocalizations can occur without a change in the shape of the excitatory tuning curve. (A)** Predicted and actual response rates of one neuron (same as in Figure 2A) to all the vocalizations in the control condition. **(B)** Predicted (blue lines) and actual responses (gray bars) to two vocalizations in the control condition. **(C)** Predicted and actual response rates to all the vocalizations in the drug condition. **(D)** Predicted (blue lines) and actual responses (gray bars) to two vocalizations in the drug condition. Symbols inset in spectrograms match those vocalizations in the scatter plots. Each symbol denotes a different vocalization.

Seventeen of the neurons with decreased selectivity (59%), had increased firing rates to tones without broadening of their excitatory frequency tuning curves. Figure 5 shows one example of a neuron with this type of response. The effects of inhibition on responses to pure tones for this neuron are shown in Figure 2A. Figure 5A plots the response rate predicted based on the excitatory frequency tuning curve of the neuron to each of the 14 vocalizations (each vocalization is a different symbol) vs. the actual response of the neuron under control conditions. This neuron did not respond to 8 of the 14 vocalizations in the control condition (SI = 0.57). Figure 5B shows the fit of the excitatory tuning curve prediction to the actual response for two of the vocalization stimuli. The symbol that is inset in the spectrogram matches the symbol in Figure 5A. The neuron did not respond to the first displayed vocalization (USV10) with enough spikes to be considered a response (spikes to less than 50% of presentations). The predicted response based on the excitatory tuning curve matched the actual response reasonably well; both the predicted and actual responses were below our threshold for a response. The neuron responded to the second displayed vocalization (USV12) as was predicted based on the excitatory frequency tuning curve.

Figure 5C shows the predicted responses to vocalizations vs. the recorded responses in the drug condition. The predicted and actual response rates were higher for each of the vocalization stimuli under the drug condition compared to the control condition. Selectivity also decreased. The SI value was 0.36 in the drug condition compared to 0.57 in the control condition. The fit of the response rates predicted from the excitatory tuning curve to the actual response rates to two of the vocalizations are shown in Figure 5D. In each of the plots, the response rate to the vocalizations was higher than in the control condition, and the neuron responded to the USV10 vocalization. Based on the frequency tuning profile of this neuron (Figure 2A), it is clear that the change in selectivity was not due to a change in shape of the excitatory frequency tuning curve.

**Figure 6:**
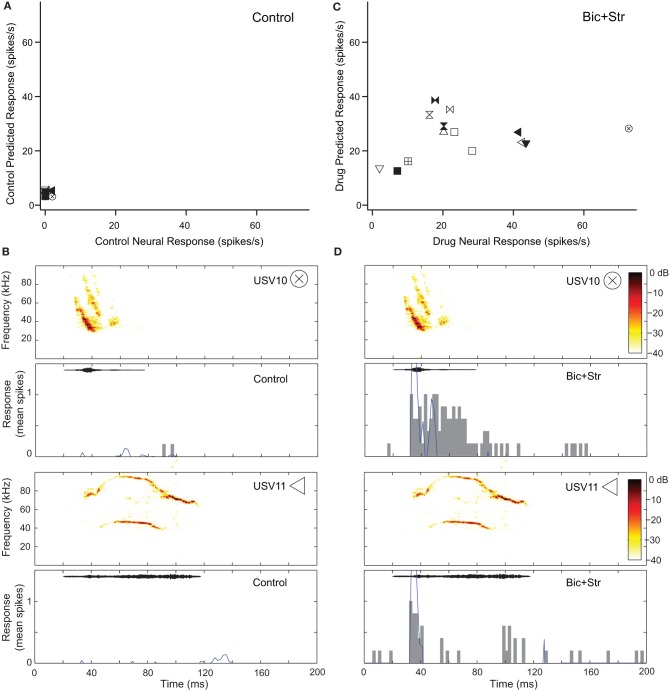
**Decreased selectivity to vocalizations can occur with a change in the shape of the excitatory frequency tuning curve. (A)** Predicted and actual response rates of one neuron (same as in Figure 2B) to all the vocalizations in the control condition. **(B)** Predicted (blue lines) and actual responses (gray bars) to two vocalizations in the control condition. **(C)** Predicted and actual response rates to all the vocalizations in the drug condition. **(D)** Predicted (blue lines) and actual responses (gray bars) to 2 vocalizations in the drug condition. Symbols inset in spectrograms match those vocalizations in the scatter plots. Each symbol denotes a different vocalization.

Blocking GABA_A_R and GlyR increased response rate and broadened the V-shaped, excitatory frequency tuning curves of 10 neurons that showed decreases in selectivity (34%). Figure 6 shows this effect for the neuron whose responses to pure tones are shown in Figure 2B. This neuron did not respond to any of the vocalizations in the control condition (Figure 6A). Figure 6B shows the fit of the excitatory frequency tuning curve prediction and the actual response for two of the vocalizations. In both cases, the neuron did not respond to the vocalization because the spectral content of the vocalization did not fall within the excitatory frequency tuning curve. As shown in Figure 6C, blocking GABA_A_R and GlyR decreased the selectivity of the neuron dramatically. The neuron responded to 11 of the vocalizations in the drug condition. Blocking inhibition broadened the excitatory frequency tuning curve (Figure 2B) resulting in an increased number of vocalizations that had spectral content within the excitatory frequency tuning curve. This can be seen for two of the vocalizations (Figure 6D) where the predicted response based on the excitatory frequency tuning curve is greater than in the control condition.

**Figure 7:**
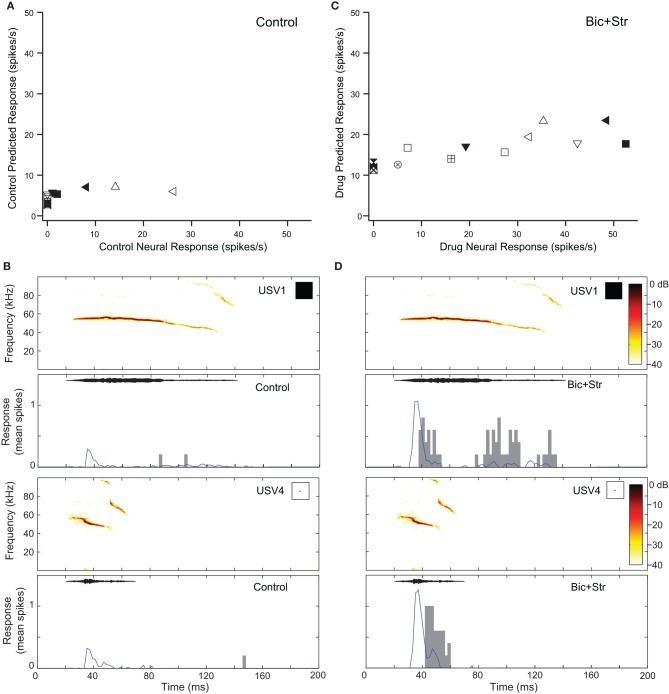
**Decreases in selectivity can occur in O-shaped neurons. (A)** Predicted and actual response rates of one neuron (Figure 2C) to all the vocalizations in the control condition. **(B)** Predicted (blue lines) and actual responses (gray bars) to two vocalizations in the control condition. **(C)** Predicted and actual response rates to all the vocalizations in the drug condition. **(D)** Predicted (blue lines) and actual responses (gray bars) to two vocalizations in the drug condition. Symbols inset in spectrograms match those vocalizations in the scatter plots. Each symbol denotes a different vocalization.

Blocking GABA_A_R and GlyR increased response rate and changed O-shaped frequency tuning curves into V-shaped tuning curves in two of the neurons that showed decreases in selectivity (7%). Figure 7 shows the responses of one of those O-shaped neurons (same neuron as Figure 2C) to vocalizations in the control and drug conditions. In the control condition, this neuron had a SI value of 0.78 (no response to 11 of the vocalizations; Figure 7A). Figure 7 shows the predicted and actual responses for two vocalizations. The neuron was not predicted to respond to either of the displayed vocalizations (USV1 and USV4) with a rate greater than the criteria for defining an evoked response, and the neuron did not respond to these stimuli. Blocking GABA_A_R and GlyR decreased the selectivity of the neuron (Figure 2C). The SI of the neuron decreased to 0.36. With a change from an O-shaped to a V-shaped tuning curve, the excitatory tuning curve predicted the neuron would respond to many more vocalizations and the recorded responses matched these predictions well (Figure 7D).

**Figure 8:**
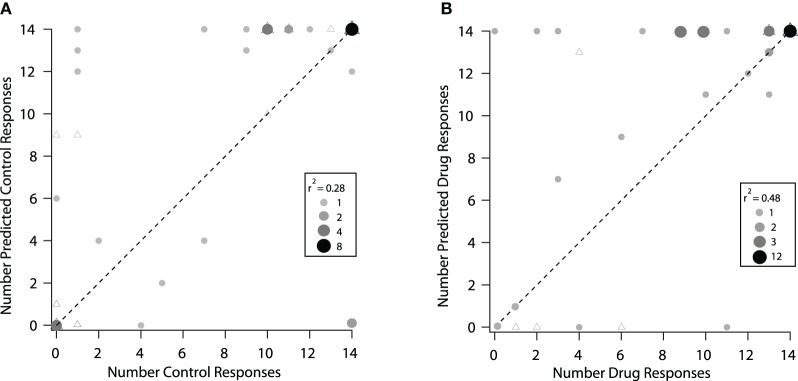
**Predicted responses to vocalizations based on the excitatory frequency tuning curves were better under drug conditions. (A)** Control condition. The predicted number of vocalizations to evoke a response is plotted vs. the actual number that evoked a response for each neuron. **(B)** Same neurons are plotted under the drug condition. The triangles represent neurons that showed a change in rate and a change in the shape of their excitatory frequency tuning curve and circles represent neurons that showed a change in rate only within the original tuning curve. In some cases, the triagles are covered by the circles. The shade and size of the circle represent the number of neurons overlapping at that point (if a triangle is underneath, it is counted and represented in the size of the circle). The size of the point and the shade of gray indicates the number of neurons that are represented by the particular dot in each of the control and drug plots. The dashed line represents equality in the number of predicted and actual responses.

The modeling techniques we used here help us to understand how changes in the excitatory frequency tuning curves when inhibition was blocked were related to changes in selectivity to vocalizations in the IC. In Figure 8 we compare how well the excitatory tuning curves predicted the actual responses in the control and drug conditions for 40 neurons (the set of neurons presented with vocalization stimuli that had tuning curve data). In the control condition, the number of vocalizations predicted to evoke responses was greater than the number of vocalizations that actually evoked responses in the majority (19/40) of neurons. For example, there were six neurons that were predicted to respond to four or more vocalizations, but either only one or none of the vocalizations evoked responses (Figure 8A). For 14 neurons, the predictions matched the recorded responses and for seven neurons, the number of vocalizations predicted to evoke responses was less than the actual number that evoked responses (Figure 8A). The correlation coefficient under control conditions was *r*^2^ = 0.28. Thus, the excitatory frequency tuning curve accurately predicted responses to vocalizations in only 14/40 neurons.

When inhibition was blocked, there was a better match between the number of vocalizations predicted to evoke responses and the number that actually evoked responses, as shown by the higher correlation coefficient (*r*^2^ = 0.48). In the drug condition there were 17 neurons where the predictions matched the recorded responses (Figure 8B). A big difference was that with inhibition blocked there was now only one neuron that did not respond to any vocalizations when it was predicted to respond to many. It is clear, however, that even when inhibition was blocked, the predictions for some neurons remained poor. In some cases, more vocalizations were predicted to evoke responses than actually did, and in other cases, the neurons responded to one or more of the vocalizations even though they were not predicted to respond.

Figure 8 also identifies the neurons that had changes in the shape of their tuning curve vs. those that only had changes in rate within the original frequency tuning curve. There was no obvious difference in the predictions for these groups of neurons. This suggests that inhibition was acting in multiple ways on these neurons to create selectivity to vocalizations.

## Discussion

In this study we examined how inhibition in the IC modulates responses to simple and complex sounds in awake mice. We found that inhibitory inputs modulate responses to simple stimuli by modulating firing rate, shaping frequency tuning curves in a variety of ways, and altering temporal firing patterns. We also found that inhibitory inputs increase selectivity to vocalizations, but in a diverse manner. Thus, the complex interplay between excitation and inhibition in the IC is important for creating the diversity of response properties in the IC.

### Inhibitory inputs modulate responses to simple stimuli in the IC of awake mice

Many studies have tested the role of inhibition in regulating response properties in the IC (Faingold et al., 1989, 1991; Vater et al., 1992a; Yang et al., 1992; Park and Pollak, 1993, 1994; Casseday et al., 1994; Klug et al., 1995; Fuzessery and Hall, 1996; Le Beau et al., 1996; Palombi and Caspary, 1996; Burger and Pollak, 1998; Koch and Grothe, 1998; LeBeau et al., 2001; Caspary et al., 2002; Zhang and Kelly, 2003). To our knowledge, ours is the first study to examine the role of inhibition in modulating responses to simple and complex stimuli in the IC of awake mice. We anticipated that our results for simple stimuli would be the same as previously reported for other mammalian species under anesthesia and for awake bats, however, it was nonetheless important to determine this explicitly since mice are becoming an important model for understanding neural mechanisms of auditory processing disorders due to the benefits of genetic engineering.

In general, we found that the effects of locally blocking GABAergic and glycinergic receptors by applying receptor antagonists in the IC of mice are the same as have been reported in other species. We describe these effects here, and also propose different microcircuits that could contribute to the effects we observed. Our techniques in this study do not allow us to ascribe specific circuits to particular effects of blocking inhibition, but we can use these putative circuits for future testing of specific hypotheses to provide further insight into the mechanisms of creating heterogeneous and selective responses in the IC. Although we propose particular microcircuits to describe particular effects of blocking inhibition, it is highly likely that multiple circuits contribute to an individual neuron's responses to simple and complex sounds. In addition, it is highly likely that the microcircuitry is different in each neuron, thus contributing to the diversity of response properties in the IC.

**Figure 9:**
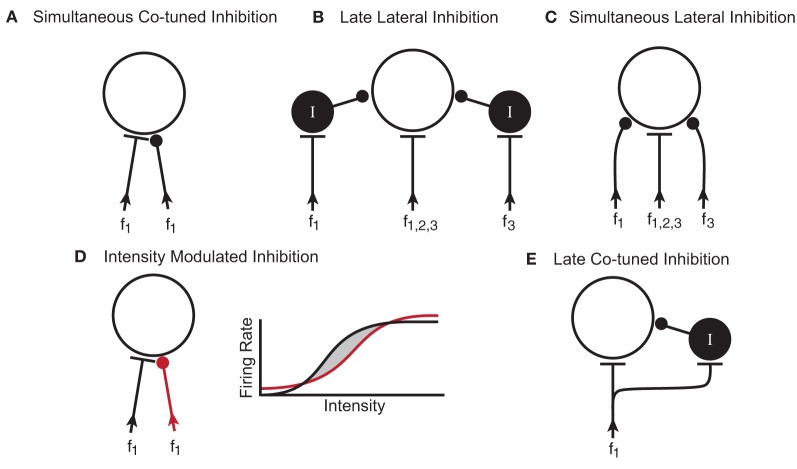
**Potential microcircuits that could shape responses to simple and complex stimuli in the IC. (A)** Simultaneous co-tuned inhibition. The excitatory and inhibitory inputs have similar frequency tuning and arrival time at the IC neuron. **(B)** Late lateral inhibition. Lateral inhibition results from inhibitory interneurons (I) with frequency ranges that are lateral (f1 and f3) to the excitatory input (f1,2,3). Synapsing onto an interneuron could delay the arrival time of the inhibition relative to the excitatory input. **(C)** Simultaneous lateral inhibition. Lateral inhibition results from direct inhibitory inputs (f1 and f3). **(D)** Intensity modulated inhibition. Excitatory (black) and inhibitory (red) inputs are co-tuned in frequency but have different rate-intensity responses. The rate-intensity function shows that at low and high intensities, the inhibitory input (red line) has a greater response than the excitatory input (black line). At middle intensities, the excitatory input has a greater response than the inhibitory input and the neuron fires (shaded region between the black and red lines). **(E)** Late co-tuned inhibition. Excitatory inputs synapse directly onto the IC neuron and onto an inhibitory interneuron such that the late arrival time of the inhibitory input creates an onset response. In all diagrams f, frequency; I, inhibitory interneuron.

The ubiquitous effect of blocking GABA_A_R and GlyR in the IC of awake mice was an increase in response rate. In three of the neurons, the firing rate increases were only in spontaneous activity, and not evoked activity. In about half of the neurons, the increase in tone-evoked response rate was not accompanied by a change in the shape of the excitatory frequency tuning curve. One interpretation of this is that the excitatory and inhibitory inputs in these neurons were co-tuned. A suggested microcircuitry to create this response is shown in Figure 9A, where the inhibitory inputs are aligned in frequency with the excitatory inputs (Kelly and Caspary, 2005) and arrive simultaneously. In this microcircuitry, the inhibition only decreases response rate within the frequency range of the excitatory inputs. In another potential circuit, the inhibition could be un-tuned in frequency. This circuitry could explain how spontaneous activity increases when inhibitory receptors are blocked.

In about 50% of the neurons, blocking inhibitory receptors changed the shape of their excitatory frequency tuning curve. This expansion of the tuning curve could occur on both the high and low frequency sides, but more often there was a large expansion on the low frequency side (Yang et al., 1992; LeBeau et al., 2001). One interpretation of these results is that GABAergic and/or glycinergic inputs onto IC neurons create lateral inhibition. The two circuit diagrams illustrated in Figures 9B,C are potential ways that inhibitory inputs to IC neurons could narrow excitatory frequency tuning curves. In both putative circuits, the inhibitory inputs are aligned with the excitatory input but have a broader frequency range. In one circuit (Figure 9B), the inputs coming into the IC are all excitatory and some of them synapse onto GABAergic interneurons. Considering that between 20–40% of the neurons in the IC are thought to be GABAergic (Caspary et al., 1990; Oliver et al., 1994; Winer et al., 1996), it is likely these interneurons play a role in shaping responses. In the second putative circuit (Figure 9C), the inhibitory inputs arise from lower brainstem nuclei and synapse directly on the IC neuron. In the current study, we are unable to distinguish between these two potential microcircuits but it is probable that both occur, either in the same neuron or in different neurons.

Another way that inhibition can modulate the shape of the tuning curve is to create O-shaped responses, where high intensity stimuli inhibit the neural response. The circuit diagram in Figure 9D illustrates one way inhibition could do this. In this case, excitatory and inhibitory inputs to the IC neuron are co-tuned in frequency and occur simultaneously, but they have different rate-intensity responses. The rate-intensity plot in Figure 9D shows that at low and high intensities the inhibitory input has a greater response (red line) and the neuron does not fire. At middle intensities (shaded region between the red and black lines), the excitatory input (black line) has a greater response and the neuron fires.

Besides shaping response rate and shape of the excitatory frequency tuning curve, inhibitory inputs to IC neurons can also affect temporal firing properties. Changes in latencies to monaural and binaural stimuli have been observed in both the mustached bat and guinea pig after iontophoretically blocking inhibitory receptors in IC (Park and Pollak, 1993; Le Beau et al., 1996). The changes in latency observed in our study (5–105 ms) agree with those seen in the mustached bat where the reported changes in latency spanned from 5 to 65 ms. Inhibitory inputs to IC neurons may lengthen latency by either arriving earlier or simultaneously with excitatory inputs such that an increased amount of time is required for the neuron to reach threshold (Park and Pollak, 1993).

Inhibition can also shape the temporal firing patterns of neurons. Onset responses become more sustained in some IC neurons when GABA_A_R and GlyR are blocked (Le Beau et al., 1996; Jen and Zhang, 1999). Figure 9E shows a putative circuit diagram to explain how inhibitory inputs to IC neurons could create onset responses to tonal stimuli. In this case, the excitatory input synapses directly on the recorded IC neuron as well as onto an inhibitory interneuron. The longer delay of the inhibitory input suppresses the evoked response after the onset thus creating a phasic response.

It is clear from *in vivo* studies where inhibitory receptors can be pharmacologically blocked that inhibition plays a role in shaping the frequency and temporal responses of IC neurons to simple stimuli. The sources of input to these neurons however is harder to determine in these types of studies. Inhibitory projections to the IC come from various brainstem nuclei such as the nuclei of the lateral lemniscus (Adams and Mugnaini, 1984; Gonzalez-Hernandez et al., 1996; Kelly and Li, 1997; Vater et al., 1997; Zhang et al., 1998), the superior paraolivary nucleus (Helfer et al., 1989; Kelly and Li, 1997; Kulesza et al., 2003), and the lateral superior olive (Saint Marie et al., 1989; Glendenning et al., 1992). Inhibitory inputs to IC also arise from the medial geniculate body (Vater et al., 1992b) and perhaps the auditory cortex (Adams, 1979; Jen et al., 2001). Moreover, inhibitory interneurons likely contribute to shaping IC response properties. The various sources of inhibitory inputs with their own unique response properties likely contribute to creating many different microcircuits in the IC that shape heterogeneous responses to simple stimuli.

### Inhibitory circuits increase selectivity to vocalizations in the IC of awake mice

Whereas the importance of GABAergic and glycinergic inhibition in shaping responses to simple, binaural and modulated sounds has been well documented (Faingold et al., 1991; Vater et al., 1992a; Yang et al., 1992; Park and Pollak, 1993, 1994; Casseday et al., 1994; Klug et al., 1995; Fuzessery and Hall, 1996; Le Beau et al., 1996; Burger and Pollak, 1998; Koch and Grothe, 1998; LeBeau et al., 2001; Caspary et al., 2002; Zhang and Kelly, 2003), the role of inhibitory microcircuitry in shaping responses to behaviorally relevant vocalizations has received less attention. Only two studies have examined the role of inhibition in shaping selectivity to vocalizations, both done in bats (Klug et al., 2002; Xie et al., 2005). In these studies, blocking inhibition decreased selectivity to social vocalizations in the IC but not in the nuclei of the lateral lemniscus suggesting that inhibition plays a role in creating selectivity to vocalizations in the IC of bats.

Our findings in the IC of awake mice are similar. Pharmacological blocking of GABAergic and glycinergic inputs to IC neurons significantly decreased neuronal selectivity to vocalizations. The magnitude of change varied across neurons. In the most extreme cases, a highly selective neuron (responding to 1 or 2 vocalizations) became completely un-selective and responded to all the vocalizations when GABA_A_R and GlyR were blocked. In addition, some neurons did not respond to any of the vocalizations under control conditions but then responded to some or all of them when GABA_A_R and GlyR were blocked. These results indicate that inhibition is important in shaping selectivity to vocalizations in the IC.

There are potentially multiple ways that inhibition could affect selectivity to vocalizations. In this study, we used the effects of inhibition on frequency tuning curves to predict the effects of inhibition on selectivity to vocalizations. While the underlying inhibitory circuitry that shapes selectivity to vocalizations cannot be specifically determined by our experimental methods, the effect of inhibition on response properties to tones and vocalizations can provide clues and stimulate future studies.

As described above, one effect of inhibition is to decrease firing rate to tones within the excitatory frequency tuning curve without changing its shape. This suggests that the inhibitory inputs (at least the ones that were pharmacologically blocked) have similar frequency tuning as the excitatory inputs and possibly keep the membrane potential at subthreshold levels for some vocalizations and not others, thereby selectively shaping responses. A limitation of our study is the use of extracellular neural recordings where subthreshold excitatory and inhibitory responses cannot be measured. To better understand how inhibitory microcircuitry is operating at subthreshold levels, future work should include measuring synaptic currents in responses to vocalization stimuli with *in vivo* whole cell recordings. These sorts of future studies would help tease apart how the thresholds, latencies, durations, and strengths of multiple inhibitory inputs to each IC neuron likely create the heterogeneous responses to vocalizations that occur in the IC (Klug et al., 2002; Holmstrom et al., 2010).

Another way that inhibition could create selectivity to vocalizations is by sharpening the excitatory frequency tuning curve so that fewer vocalizations contain energy that falls within the excitatory region. Moreover, by creating neurons with inhibitory side-bands, selectivity to vocalizations can be enhanced because any vocalization that contains spectral energy within these inhibitory bands would not evoke a response. Therefore, in the case where two vocalizations share spectral content that falls within the excitatory tuning curve but one also has energy that falls within the inhibitory side-bands, the neuron will not respond to the one that stimulates the inhibition, thereby being selective. Thus, neurons with inhibitory side-bands could be more selective to vocalization stimuli compared to those neurons with just a narrow excitatory region. This is a mechanism also proposed for creating selective responses to vocalizations in the mustached bat IC (Portfors, 2004). Less than 40% of the neurons in our study that showed decreased selectivity to vocalizations had expanded tuning curves when inhibition was blocked. This could mean that only about half of the neurons in the IC of awake mice have inhibition side-bands, that the side-bands of some neurons were narrower than the 2 kHz resolution that we tested here, or that we did not completely block the inhibition in some neurons. One study in anesthetized mice suggested that the majority of neurons in IC have inhibitory side-bands (Egorova et al., 2001). It is unclear whether the differences in the two studies is due to anesthesia or methods. Regardless of the extent of inhibitory side-bands in IC neurons, it is clear that inhibitory circuitry that creates side-bands is important for creating selectivity to vocalizations in some neurons.

Inhibitory microcircuits in IC can modify the shape of excitatory frequency tuning curves in other ways that can shape responses to vocalizations. For example, neurons with O-shaped tuning curves, where responses are suppressed to stimuli at high intensities, show selectivity to vocalizations. In these neurons, a vocalization would need to have the appropriate spectral content as well as intensity profile to evoke a response. We found that 15% of neurons in awake mouse IC had O-shaped tuning curves, and that this type of tuning plays a role in creating selectivity to vocalizations. The distribution of O-shaped neurons seems to depend on the species and the recording method. In decerebrate cats, O-shaped neurons are common, comprising about 50% of neurons in the central nucleus of the IC (Ramachandran et al., 1999) but they are less common (5–10%) in non-decerebrated cats (Ehret and Merzenich, 1988) and mice (Egorova et al., 2001). Regardless, the inhibitory microcircuitry that creates these responses is likely important in shaping responses to vocalizations to, at least, a small extent in mice.

Our modeling results, using the frequency tuning curve to predict responses to vocalizations, are similar to those obtained by Klug et al. (2002) in the IC of Mexican free-tailed bats. They also found that responses to vocalizations are not well predicted from responses to tones, and that blocking inhibition pharmacologically as we did here, decreases selectivity and improves predicted responses based on tones. In both studies, however, predictions for some neurons remained poor when inhibition was blocked. In some neurons, more vocalizations were predicted to evoke responses than actually did, and in other neurons, the model failed to predict responses to vocalizations that actually evoked responses. These findings suggest the presence of other mechanisms for creating selectivity to vocalizations. It is well known that there are multiple non-linearities in the IC as well as in brainstem nuclei, and these likely are involved in creating selectivity to vocalizations. For example, combination sensitivity is important for creating selectivity to vocalizations in bats (O'Neill and Suga, 1979; Mittmann and Wenstrup, 1995; Portfors and Wenstrup, 1999; Portfors, 2004) and these types of responses have also been found in mice (Portfors and Felix II, 2005). Other non-linearities that occur in lower brainstem nuclei (Spirou et al., 1999; Portfors and Wenstrup, 2001b; Marsh et al., 2006) are also factors that likely shape selectivity to vocalizations in the IC. In addition, we have previously shown that neurons in the IC of mice that are tuned to frequencies much lower than the spectral content of the vocalization respond to these vocalizations because of cochlear distortions (Portfors et al., 2009). These neurons may be the ones that respond to particular vocalizations even though they are not predicted to based on single tone frequency tuning curves. In general, multiple mechanisms throughout the ascending auditory system contribute to creating the diversity of selective responses to vocalizations in the IC.

The results found here in mice, combined with the findings in bats (Klug et al., 2002; Xie et al., 2005), provide strong evidence that inhibitory microcircuits in IC play an important role in shaping selectivity to vocalizations. That these inhibitory microcircuits are similar in mice and bats suggests that the IC has evolved common circuitry across mammals. In addition, the finding that inhibition shapes selectivity to vocalizations in mice provides further evidence that selectivity to behaviorally relevant sounds is created at the level of the auditory midbrain (Portfors and Wenstrup, 2001a; Bauer et al., 2002; Nataraj and Wenstrup, 2005; Xie et al., 2005) rather than at the auditory cortex.

### Conflict of interest statement

The authors declare that the research was conducted in the absence of any commercial or financial relationships that could be construed as a potential conflict of interest.
